# Dr. Arthur James Macdonald Bentley

**Published:** 1911-04-29

**Authors:** 


Dr. Arthur James Macdonald Bentley has died suddenly at Llandrindod Wells. He studied at Edinburgh University, where he graduated M.D., as a gold medallist, in 1889, having taken his M.R.C.S. in 1871. His appointments abroad comprised those of colonial surgeon to the Straits Settlements, medical adviser to the Johore Government, physician to the Sultan, and visiting surgeon to the Leper Hospital, Singapore. Later he practised at Cairo in the winter, and in the summer at Llandrindod Wells, where he only arrived from Egypt a few days ago. He was to have lectured last week on "Tropical Diseases" at Owens College, Manchester. His publications included "Beri Beri, its Ætiology, Symptoms, Pathology, and Treatment," and "Wintering in Egypt."

